# Solvent-Free Synthesis of Covalent Organic Frameworks for High-Performance Room Temperature Ammonia Sensing

**DOI:** 10.3390/mi17040499

**Published:** 2026-04-20

**Authors:** Jiayi Wu, Xinru Zhang, Hongwei Xue, Xiaorui Liang, Lei Zhang, Qiulin Tan

**Affiliations:** 1 State Key Laboratory of Extreme Environment Optoelectronic Dynamic Measurement Technology and Instrument, North University of China, Taiyuan 030051, China; sz202406123@st.nuc.edu.cn (J.W.); zxr13293844364@163.com (X.Z.); sz202306182@st.nuc.edu.cn (H.X.); 2 Key Laboratory of Micro/Nano Devices and Systems, Ministry of Education, North University of China, Taiyuan 030051, China; 20250015@nuc.edu.cn

**Keywords:** covalent organic frameworks, solvent-free synthesis, flexible sensing, ammonia gas sensor

## Abstract

High-sensitivity rapid detection of ammonia (NH_3_) in environmental monitoring, industrial safety, early diagnosis, and other fields is of great significance. Covalent organic frameworks (COFs) have shown great potential in the field of gas sensing due to their designable porous structure and active sites. However, the traditional solvothermal synthesis method of COFs has problems such as cumbersome steps, high energy consumption and serious environmental pollution. Therefore, it is of great significance to invent a new method for COF synthesis that is green and efficient and makes it easy to conduct flexible ammonia gas sensing. This study first reported a solvent-free synthesis of imine connection 1,3,5-Triformylbenzene (TFB) and p-Phenylenediamine (PDA)—a new strategy for COF. This method innovatively employs zinc trifluoromethyl sulfonate (Zn(OTf)_2_) as a bifunctional catalyst. This catalyst not only efficiently catalyzes para-phenylenediamine, but its zinc ions also play a unique structural guiding role, guiding the reactants to be arranged in a directional manner, thereby constructing a highly ordered porous crystal structure. A series of characterizations confirmed that the obtained TFB-PDA-COF had good crystallinity and a high proportion of imine bonds (C=N). The powder material was coated onto a flexible polyimide (PI) substrate, successfully constructing a resistive ammonia gas sensor that operates at room temperature. The test results show that this sensor has a high response value, rapid response/recovery capability, and good selectivity for ammonia gas. More importantly, based on a flexible PI substrate, the device can maintain stable sensing performance even under repeated bending conditions, demonstrating its great potential in practical flexible electronic applications. This work not only provides a brand-new “zinc ion-guided” paradigm for the green and controllable synthesis of COF but also lays a material foundation for their application in the next-generation flexible sensing field.

## 1. Introduction

Developing novel sensing materials and devices that offer superior performance, are simple to fabricate and can be produced on a large scale, thereby creating practical, comprehensive solutions, is a key challenge currently facing interdisciplinary fields such as materials science, analytical chemistry and sensing engineering [1,2,3,4]. In the field of industrial safety, for ammonia, as an important chemical raw material and refrigerant, trace leakage monitoring during production, storage and transportation is an essential requirement for preventing major safety accidents and safeguarding personnel’s health [5,6]. Long-term exposure to ammonia may lead to chronic health problems, such as bronchiectasis, pulmonary fibrosis, chronic respiratory irritation and impaired lung function [7,8]. Developing new sensing technologies and materials that can meet the high-performance detection requirements in these complex scenarios has become a common challenge across multiple disciplines.

Covalent organic frameworks (COFs) are a class of crystalline porous materials formed by covalent bonds between light elements (C, H, O, N, etc.). They were first reported in 2005 [9] thanks to their adjustable aperture structure, rich active sites, excellent chemical stability, and the ability to design surface functionalization. They show great application potential in fields such as gas storage, catalysis, separation and sensing [10,11,12,13,14,15]. The most typical type of COF is the imine-bond-linked COF. The structure of the imine bond (-C=N-) in it not only endows the material with high chemical and thermal stability but is also formed by the reaction of the amine (-NH_2_) group with the aldehyde (-CHO) group. It also provides modifiable active sites, enabling it to demonstrate significant potential in the fields of gas adsorption and sensing [16,17,18,19]. In the field of ammonia gas sensing, the nitrogen atoms in imines can interact specifically with ammonia gas molecules through lone pairs of electrons, thereby achieving high selectivity and high-sensitivity detection of ammonia gas. This provides an important material basis for the development of new flexible ammonia gas sensors [20,21,22]; however, the synthesis of amino COFs still faces many challenges. The traditional preparation method—solvothermal method—takes a long time (three days to a week) and mostly uses strongly polar organic solvents (such as dioxane, DMF, etc.) [23,24,25]. These solvents are highly toxic, have poor environmental friendliness, and may limit the applicability of materials in certain application scenarios. Although researchers have developed emerging synthesis strategies such as microwave-assisted methods [26,27,28], mechanized methods [29,30], and fusion methods [31,32,33,34,35] in recent years, which have made significant progress in improving synthesis efficiency (such as shortening reaction time, reducing energy consumption, and decreasing solvent usage), these methods are still costly when applied to practical sensing applications. Key links such as large-scale production, precise control of crystallinity, and long-term stability of material structure are facing severe challenges, making it difficult to meet the strict requirements of batch production and high consistency of performance of sensitive materials.

Against this background, we focus on TFB and PDA, which have lower raw material costs and are more conducive to large-scale production. This work innovatively proposes a new, efficient, and green catalytic synthesis strategy for TFB-PDA-COF. The synthesis of TFB-PDA-COF primarily relies on conventional solvothermal methods, which are time-consuming and labor-intensive. These methods require large amounts of organic solvents, severely hindering the material’s industrial development [36]. The core concept of this method is to use Zn(OTf)_2_ as a strong Lewis acid catalyst, which plays a dual role in the reaction process: on the one hand, it catalyzes the formation of imine bonds; on the other hand, the released zinc ions can undergo in situ coordination with the nitrogen atoms on the initially formed imine bonds. This coordination function is similar to a “molecular template”, dynamically guiding and stabilizing the stacking and extension of two-dimensional layered structures. Based on this collaborative mechanism, this method achieved the green and efficient synthesis of TFB-PDA-COF within a few minutes, significantly reducing or even avoiding the reliance on toxic, high-boiling-point polar solvents, demonstrating excellent synthetic economy and environmental friendliness. When the obtained materials were applied to the sensing of room-temperature ammonia gas, they demonstrated excellent high-sensitivity and rapid-response characteristics. The prepared TFB-PDA-COF flexible sensor has a current response change of approximately 60% for 100 ppm NH_3_, and this sensor has excellent gas selectivity. The excellent linear response it shows in the NH_3_ range of 10 ppm to 60 ppm reveals its great potential for actual NH_3_ detection at room temperature. The key contribution of this study lies in the development of a novel, rapid and environmentally friendly synthetic strategy for COF. This method enables the preparation of structurally stable COF material under mild conditions. Room-temperature ammonia sensing tests demonstrate that the material exhibits excellent gas-sensing properties, facilitating the transformation of ammonia-sensitive materials into practical sensing devices. This work provides a valuable reference for the synthesis and application of novel COF gas-sensing material.

## 2. Experiment

### 2.1. Solvent-Free Synthesis of TFB-PDA-COF and TFPB-PDA-Polymer

Preparation of TFB-PDA-COF: 1,3,5-Benzenetricarboxaldehyde(TFB) and p-Phenylenediamine (PDA) were weighed in a molar ratio of 1:1.5, followed by the addition of an appropriate amount of zinc trifluoromethanesulfonate (Zn(OTf)_2_). For TFB-PDA-COF to exhibit crystallinity, the molar ratio of Zn(OTf)_2_ to TFB should be at least 2:1. The mixture was ground uniformly at room temperature, then transferred to an oven and heated to 120 °C. The color turned deep red within a short time. The resulting solid powder was washed with water and ethanol to obtain yellow TFB-PDA-COF powder.

Preparation of TFPB-PDA-Polymer: 1,3,5-Tris(p-formylphenyl)benzene (TFPB) and p-Phenylenediamine (PDA) were weighed at a molar ratio of 1:1.5, and zinc trifluoromethanesulfonate (Zn(OTf)_2_) was added. The mixture was ground uniformly at room temperature, then heated in an oven at 120 °C. The resulting solid powder was washed with water and ethanol to yield yellow TFPB-PDA-Polymer powder.

### 2.2. Characterization of TFB-PDA-COF and TFPB-PDA-Polymer

Powder X-ray diffraction (PXRD) measurements were performed with a Rigaku SmartLab powder X-ray diffractometer (Rigaku Corporation, Tokyo, Japan) equipped with a 3 kW Cu anode (Cu Kα radiation, λ = 1.541 Å). Fourier transform infrared (FT-IR) spectra were recorded at 25 °C by a JASCO FT/IR-4100 FT-IR spectrometer (JASCO Corporation, Tokyo, Japan). X-ray photoelectron spectroscopy (XPS) was performed on a ULVAC PHI 5000 Versa Probe II instrument (ULVAC-PHI Corporation, Chigasaki, Japan) equipped with an electron energy analyzer using mono-energetic Al Kα X-ray. Thermogravimetry analysis (TGA) was performed by Mettler–Toledo TGA/SDTA851 star system (Mettler-Toledo Corporation, Zurich, Switzerland) with a heating rate of 5.0 °C min^−1^ from 30 to 800 °C under a nitrogen atmosphere. Scanning electron (SEM) and energy dispersive X-ray (EDX) were performed using a Hitachi SU8010 field emission SEM (Hitachi Corporation, Tokyo, Japan) operated with an accelerating electron beam voltage of 25 kV and equipped with a Bruker X-Flash 6160 EDX detector (Bruker Nano GmbH Corporation, Berlin, Germany). Transmission electron microscopy (TEM) was performed using a FEI Tecnai G2 Spirit field (FEI Corporation, Hillsboro, OR, USA) emission TEM.

### 2.3. Fabrication and Measurement of Gas Sensor

Ammonia gas sensor was fabricated using a polyimide (PI) substrate (10 × 10 mm) equipped with interdigitated copper electrodes. The PI substrate was sequentially cleaned with acetone, deionized water, and ethanol, then transferred to a drying stage and dried at 70 °C to obtain clean electrodes. Subsequently, 5 mg of covalent organic framework (COF) powder was added to 1 mL of ethanol and ultrasonicated for 45 min to form a homogeneous suspension. After thorough mixing, the suspension was taken up with a pipette and drop-coated repeatedly (5 µL per cycle) to ensure coverage limited strictly to the interdigitated electrode area. This drop-coating process was performed on a hot plate and repeated for 5–15 cycles until the device resistance stabilized. The resulting device was dried at 60 °C to yield the final gas sensor. Gas sensing performance was evaluated using a photoelectric integrated test platform, comprising primarily an SA3102 electrical measurement system and an in situ measurement cell. A bias voltage of 1000 mV was applied via an R-t source measurement unit to monitor the variation in sensor resistance.

## 3. Results and Discussion

**Figure 1:**
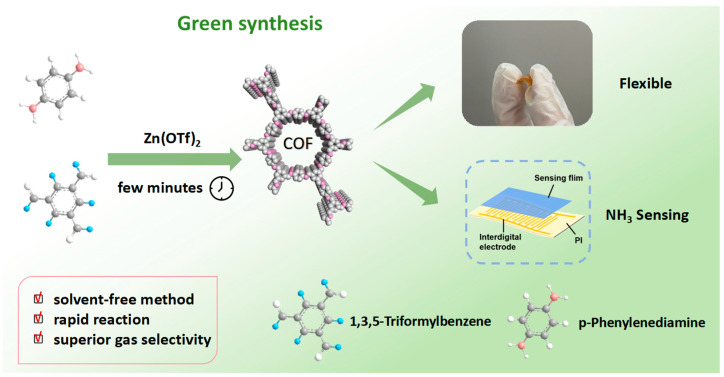
Schematic diagram of the green synthesis of TFB-PDA-COF and the preparation of a flexible ammonia sensor.

The existing literature has not paid much attention to the complex problems faced during the preparation of COFs [37,38], such as long preparation time, low crystallinity, and inevitable contact with strong polar solvents. To achieve the preparation of highly crystalline COFs with excellent ammonia gas sensing performance in a short time, this project was carefully designed. TFB and PDA were selected as the reaction monomers of COF, and zinc trifluoromethanesulfonate was used as the catalyst. After screening the preparation conditions (temperature, ratio, etc.), highly crystalline COF was finally obtained and applied in flexible ammonia gas sensing. The schematic diagram of the preparation and application of COF is shown in Figure 1.

**Figure 2:**
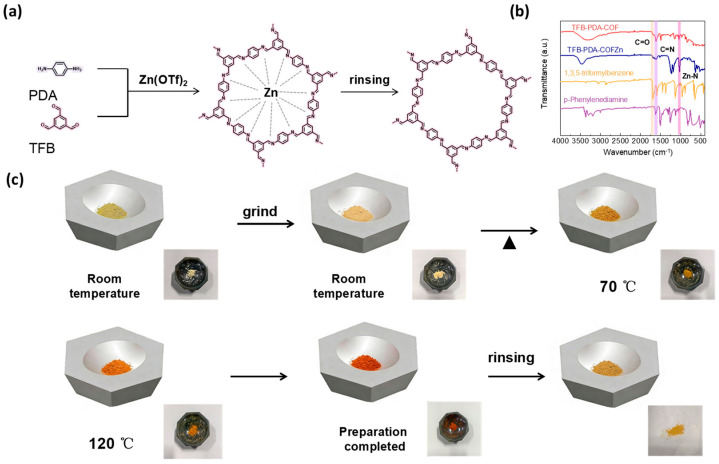
(**a**) Schematic illustration of the synthetic procedure for TFB-PDA-COF; (**b**) FT-IR spectroscopy of COF and reaction raw materials; (**c**) illustration of the color changes during the production of COF.

TFB and PDA were weighed at a molar ratio of 1:1.5, and then an appropriate amount of Zn(OTf)_2_ was added and ground together at room temperature until the mixture was uniform and turned light yellow. The mixture was then heated to 120 °C in an oven. Within a short time, the color changed to deep red. The resulting solid powder was washed to yield a yellow TFB-PDA-COF powder. The color variation observed throughout the process attracted our attention. Notably, no color changes had previously been encountered during the synthesis of imine-linked COFs, regardless of the preparation strategies employed. After the literature review, it was found that a significant color change occurs when COFs undergo metal coordination [39]. Combining the observed phenomenon and the presence of zinc in the catalyst, we speculated that the reaction process is as shown in Figure 2a. After the addition of Zn(OTf)_2_, the two monomers first form a complex of TFB-PDA-COF and zinc ions. The presence of zinc ions promotes the formation of pores, and the formed coordination bonds are relatively unstable. After water washing, they will be detached, leaving only the structure of the COF itself. To verify this hypothesis, we conducted FT-IR tests on the unreacted TFB and PDA, as well as the TFB-PDA-COFZn and the final TFB-PDA-COF formed during the process, as shown in Figure 2b. The C=O stretching vibration peak at 1696 cm^−1^ in the aldehyde group of TFB-PDA-COFZn and TFB-PDA-COF was significantly weakened compared to TFB, and a C=N stretching vibration absorption peak at 1619 cm^−1^ gradually appeared, indicating the successful completion of the Schiff base condensation reaction. Notably, a distinct Zn-N characteristic peak at 993 cm^−1^ appeared in TFB-PDA-COFZn, providing direct evidence of zinc ion-guided COF formation. To observe the entire reaction process more intuitively, the entire reaction process was photographed and recorded, as shown in Figure 2c, and the video during water washing was recorded, as shown in the video in the supporting information. The color change from red to yellow was clearly observed, providing direct evidence of the conversion from TFB-PDA-COFZn to TFB-PDA-COF.

To further demonstrate that Zn plays a guiding role in the COF-formation process, Zn(OTf)_2_ was dissolved in water to serve as a catalyst, thereby disrupting its function as a solid-state salt template. All other conditions remained unchanged from those of the solid-state reaction, and XRD analysis was performed on the products. The results are shown in Figure S1. It can be observed that when Zn(OTf)_2_ is converted from the solid to the liquid phase, the resulting product is amorphous. Furthermore, the obtained product was subjected to SEM (Figure S2) and EDS (Figure S3) analysis. SEM observations reveal a loose, stacked microstructure, while EDS analysis indicates uniform distribution of C and N elements. We divided the experimental process into four stages, unground sample, unheated sample, TFB-PDA-COFZn, and TFB-PDA-COF, and performed XRD characterization on the products at each stage, with the results shown in Figure S4. The results indicate that the typical COF characteristic diffraction peaks only appear after the Zn element has been completely removed; during the reaction process, the Zn-containing intermediates did not exhibit COF characteristic peaks. The results provide key evidence that the Zn species participates in the reaction as a catalyst and enters the COF pore channels.

**Figure 3:**
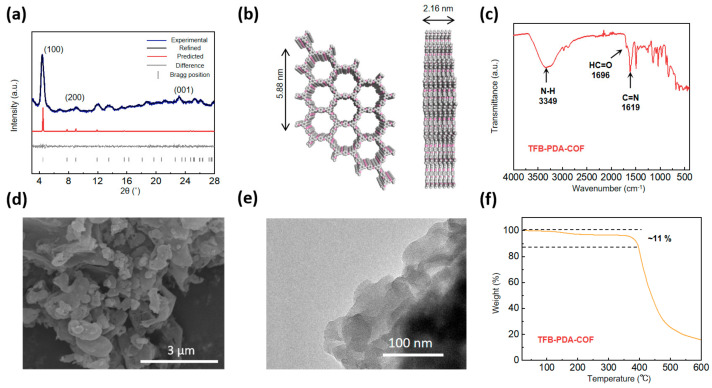
(**a**) XRD patterns; (**b**) theoretically eclipsed-AA stacking model; (**c**) FT-IR spectroscopy; (**d**) SEM; (**e**) TEM; (**f**) TGA curves of TFB-PDA-COF.

To determine that the prepared material is a COF, a series of characterizations were conducted on the obtained material, as shown in Figure 3. The powder X-ray diffraction (PXRD) pattern of TFB-PDA-COF (Figure 3a) shows a high-intensity first peak at approximately 4.37°, which can be attributed to the strong reflection of the (100) crystal plane. A peak at about 9.05° can also be attributed to the reflection of the (200) crystal plane. Similarly, the broad peak at a higher 2θ value (about 24.5°) indicates π-π stacking from the (001) crystal plane. The experimental XRD pattern is highly consistent with the simulated AA stacking model, which is in line with the results reported. We have also performed a refined analysis of the XRD data, yielding an Rwp value of 2.97% and an Rp value of 2.33%. The relatively high intensity of the (100) peak reflects the high crystallinity of these synthesized COFs. Based on the proposed model, Pawley refinement was performed on the PXRD pattern using Materials Studio 2019 to achieve full-profile fitting, and the lattice parameters of TFB-PDA-COF were obtained as a = b = 22.68 Å, c = 3.601 Å, α = β = 90°, γ = 120°. The pore size of the formed COF is approximately 1.8 nm (Figure 3b). To characterize the pore structure, BET measurements were performed on the obtained samples (Figure S5). The results show that, compared with the conventional solvothermal method, TFB-PDA-COF synthesized via the solvent-free mechanochemical method exhibits a lower BET specific surface area (7.4 m^2^ g^−1^); its pore size distribution ranges from 1.5 to 1.7 nm, which is slightly smaller than the theoretical value (~1.8 nm).

We attribute this phenomenon to the inherent characteristics of the solvent-free synthesis system. First, the absence of a solvent acting as a molecular template results in tighter interlayer π–π stacking, leading to a certain degree of lattice contraction in the framework. Additionally, amorphous components may adhere to the pore walls, collectively causing the measured pore size to be smaller than the theoretical value and the distribution to be broader. SEM characterization further reveals that the solvent-free samples consist of densely packed nanocrystals. This microstructure, characterized by the coexistence of intergranular packing voids and intragranular micropores, is also a key factor contributing to the broad pore size distribution.

Although the aforementioned structural features indicate that the pore regularity of this material is slightly inferior to that of solvothermal products, this precisely reflects the inherent nature of solvent-free kinetic-limited processes. This strategy not only avoids the use of large amounts of organic solvents but also provides a viable approach for constructing COF materials with unique microstructures. In the infrared spectrum (FTIR) test of TFB-PDA-COF (Figure 3c), the significant weakening of the C=O stretching vibration peak of the aldehyde group at 1696 cm^−1^, the obvious C=N stretching vibration absorption peak at 1619 cm^−1^, and the significant weakening of the N-H peak at 3349 cm^−1^ all indicate the successful completion of the Schiff base condensation reaction. The surface morphology of TFB-PDA-COF was characterized by scanning electron microscopy (SEM), as shown in Figure 3d. Compared to the reaction system using an aqueous solution of Zn(OTf)_2_ as a catalyst, this system exhibits a more compact and distinct layered stacking structure, strongly validating the effectiveness of the salt-templating strategy. SEM analysis of TFB-PDA-COFZn (Figure S6) also reveals a distinct layered structure, further confirming that differences in the reaction system have a significant impact on the morphology of the product. The COF synthesized by this method has a typical COF morphology, presenting a short rod-like shape at the micrometer level, formed by the stacking of short rods. The EDS spectra in (a–c) of Figure S7 show that carbon and nitrogen elements are uniformly distributed on the surface, revealing the high homogeneity of the chemical structure of TFB-PDA-COF, compared to TFB-PDA-COFZn (Figure S8), the signal for zinc has significantly weakened, indicating that the zinc has been completely removed. TEM analysis (Figure 3e) indicates that the obtained material shows a clear layered stacking morphology, but no continuous atomic-level lattice fringes were observed in the high-resolution image. This phenomenon can be attributed to the extreme sensitivity of COF materials (especially imine bonds) to high-energy electron beams. The electron beam irradiation in conventional TEM observations easily leads to local structural amorphization, thereby obscuring the intrinsic lattice information. This is also attributed to the poor intrinsic conductivity of TFB-PDA-COF; its structure is extremely unstable under high voltage, as evidenced by the rapid movement of the samples under high-voltage irradiation. Nevertheless, the appearance of the layered contour confirms the successful construction of a mid-range ordered structure dominated by π-π stacking, which is consistent with the results of XRD, FT-IR, etc., and jointly reveals the unique structural state of the material. The thermogravimetric (TGA) analysis (Figure 3f) indicates that TFB-PDA-COF has excellent thermal stability, with a mass loss of only 11% from room temperature to 400 °C. Further, elemental analysis by XPS was conducted (Figure S9). The C element was deconvoluted, with the peak at 286.2 eV being the C=N peak, which is direct evidence of the successful reaction (Figure S9a). The peak at 284.8 eV is the C-C/C-H peak on the benzene ring, and the peak at 288.6 eV is the C=O peak of unreacted TFB. Meanwhile, the N element was deconvoluted (Figure S9b), with the obvious peak at 398.8 eV being the C=N peak, and the peak at 401.8 eV being the N-H peak of incompletely reacted PDA. No distinct coordination peaks for Zn and N were observed, indicating that zinc is not present in the final TFB-PDA-COF sample.

**Figure 4:**
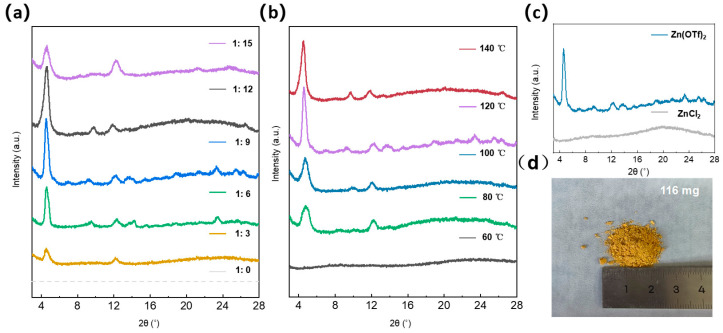
Comparative XRD images for (**a**) catalysts with different ratios; (**b**) Varying temperatures; (**c**) Different catalysts; (**d**) Schematic illustration of large-scale production of COF.

During the material preparation process, the temperature and the proportion of the catalyst have a significant impact on the crystallinity. Therefore, a series of control experiments were conducted to explore the optimal preparation process. Firstly, with the temperature remaining constant, control experiments with different catalysts were carried out, and the crystallinity was determined by XRD patterns (Figure 4a). The ratios of TFB and Zn(OTf)_2_ were 1:0, 1:3, 1:6, 1:9, 1:12, and 1:15. As shown in Figure 4a, when the ratio was 1:0, no product was observed, indicating that the presence of the catalyst is a necessary condition for the formation of COF. It was also observed that when the catalyst content was in the range of 1:6 to 1:12, the (100) crystal plane had a relatively narrow half-peak width, indicating the formation of high crystallinity. As the catalyst content further increased, the crystallization peak of the (100) crystal plane began to broaden, suggesting a decrease in crystallinity. To quantitatively analyze changes in crystallinity, the half-width at half-maximum (HWHM) of the (100) crystal plane was calculated. As shown in Table S1, crystallinity actually decreased as the salt ratio increased further. This is attributed to an insufficient amount of monomer and an excess of catalyst in the reaction system, collectively demonstrating that a higher catalyst loading does not necessarily yield better results. Secondly, with the catalyst proportion remaining constant, tests were conducted at different temperatures, and the crystallinity was still determined by XRD patterns (Figure 4b). The selected temperatures were 60 °C, 80 °C, 100 °C, 120 °C, and 140 °C. When the temperature was 60 °C, no obvious crystallization peak was observed, indicating an amorphous state, proving that a certain temperature requirement is necessary for the formation of COF. When the temperature was above 80 °C, a distinct crystallization peak appeared. When the temperature was above 120 °C, the (100) crystal plane had a relatively narrow half-peak width, indicating the formation of highly crystalline COF. At the same time, we conducted a quantitative analysis of the (100) crystal plane. As shown in Table S2, temperature played a critical role in the success of the reaction. Following a series of experiments comparing different catalysts and temperatures, we determined the optimal conditions for the preparation of TFB-PDA-COF using this method.

To systematically investigate the effect of Lewis acid strength on the crystallinity of the products, this study not only selected zinc trifluoromethanesulfonate (Zn(OTf)_2_) as a strong Lewis acid catalyst but also introduced zinc chloride (ZnCl_2_) which exhibits relatively weaker Lewis acidity, for comparative experiments. The crystallinity of the obtained products was using X-ray diffraction (XRD) patterns (Figure 4c). It is worth noting that in the reaction system using zinc chloride as the catalyst, the XRD pattern did not exhibit distinct sharp diffraction peaks but rather broad, diffuse peaks. This result indicates that, compared to a strong Lewis acid environment, weak Lewis acid conditions are not conducive to the formation of long-range ordered crystal structures, suggesting that the successful formation of crystals is subject to a certain threshold of dependence on the Lewis acid activity of the catalyst. After a series of control experiments, COF was successfully synthesized and scaled up to the milligram level (Figure 4d), achieving extremely easy batch synthesis.

**Figure 5:**
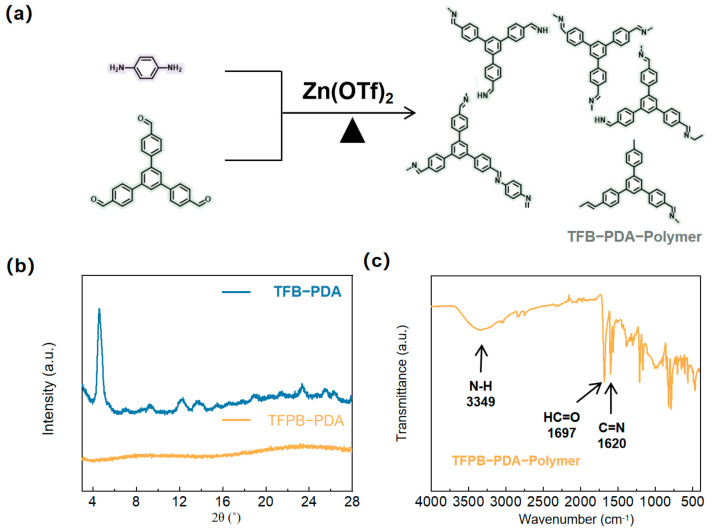
(**a**) Schematic illustration of the synthetic procedure for TFPB-PDA-Polymer; (**b**) XRD patterns; (**c**) FT-IR spectroscopy of TFPB-PDA-Polymer.

Meanwhile, the coordination bond formed by zinc ions and nitrogen elements is metastable and can be easily removed after water washing. It can be observed that the strength of the coordination bond may weaken as the COF pore size increases. When the pore size is further enlarged, the guiding ability of zinc ions will be greatly weakened. Therefore, it is speculated that the coordination mode guided by zinc ions may be limited to the small-pore TFB-PDA-COF. To verify this conjecture, the monomers were replaced with 1,3,5-Tris(p-formylphenyl)benzene (TFPB) and PDA, and the reaction was carried out under the catalysis of Zn(OTf)_2_. The experimental flow chart is shown in Figure 5a. The structure was analyzed. The XRD spectrum (Figure 5b) did not show obvious crystalline peaks, but the infrared (FT-IR) spectrum (Figure 5c) showed a distinct C=N stretching vibration absorption peak at 1620 cm^−1^ and a significant weakening of the N-H peak at 3349 cm^−1^, indicating the successful completion of the Schiff base condensation reaction. However, it is worth noting that there is still a distinct C=O stretching vibration peak of the aldehyde group at 1697 cm^−1^, indicating that the reaction was not completely carried out and some monomers were retained, which may affect the performance of the sensor.

**Figure 6:**
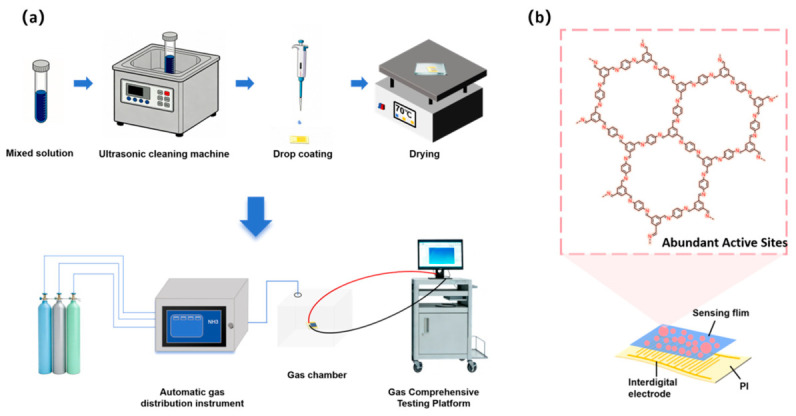
(**a**) Flowchart of Preparation and Testing of Ammonia Gas Sensor; (**b**) Structural Formula of Composite Materials for Ammonia Gas Sensors.

Through the experimental characterization and mechanism research of the above-mentioned system, this project successfully developed a new solvent-free synthesis strategy based on zinc ion guidance, achieving rapid and green preparation of highly crystalline TFB-PDA-COF. The comprehensive analysis results of XRD, FT-IR, XPS and SEM not only confirmed that the material has a regular imine-bonded framework, good crystal structure and unique micro-morphology but also deeply revealed the dual role mechanism of zinc ions in catalyzing Schiff base condensation and guiding the ordered assembly of the structure. Based on this material, a high-performance flexible ammonia gas sensor was further constructed. The schematic diagram of its preparation process is shown in Figure 6a, and the specific preparation process is described in the Supplementary Materials. The microscopic schematic diagram of the coating and forming of its COF active layer on the flexible substrate is shown in Figure 6b.

To investigate the response characteristics of covalent organic frameworks to ammonia, the researchers used a sensor testing platform equipped with an automatic gas delivery system (Figure S10) to assess gas sensing performance. During testing, the sensors were mounted within a gas chamber, and an NH_3_/air mixture was introduced via an automatic gas mixer to regulate the concentration of ammonia. A schematic diagram of the TFB-PDA-COF material coated onto a flexible substrate is shown in Figure S11. First, the researchers conducted a comparative analysis of the response performance of the two materials.

**Figure 7:**
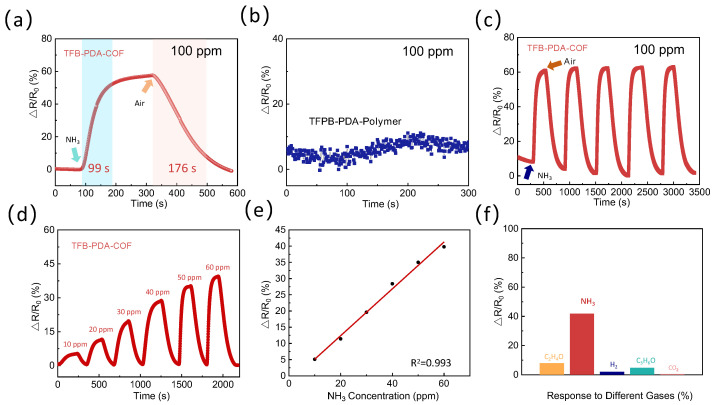
(**a**) Response and recovery time of ammonia gas sensor at 100 ppm of TFB-PDA-COF; (**b**) response and recovery time of ammonia gas sensor at 100 ppm of TFPB-PDA-Polymer; (**c**) cyclic Performance of TFB-PDA-COF at 100 ppm; (**d**) dynamic response profiles of ammonia sensors in the 10–60 ppm NH_3_ concentration range; (**e**) the relationship between temperature and response value; (**f**) the dynamic response curves of the ammonia sensor to different gases at a concentration of 50 ppm.

Figure 7a,b show the response changes in TFB-PDA-COF and TFPB-PDA-Polymer when exposed to 100 ppm NH_3_ at 25 °C. Under 100 ppm, the response time of TFB-PDA-COF is approximately 99 s, the recovery time is 176 s, and the response value is about 60%, which has certain advantages over the reported COF and MOF-based ammonia sensors, as shown in Table S3. However, the response of TFPB-PDA-Polymer is extremely weak and fluctuates greatly due to insufficient active groups (-NH_2_-) and incomplete reaction under these conditions, making it impossible to analyze the response time and recovery time. The behavior of TFB-PDA-COF can be attributed to the adsorption of ammonia molecules on the surface of COF, which provides electrons to the material, thereby increasing the electron concentration and reducing the resistance. Notably, COF exhibits a higher response rate (60% at 100 ppm) and excellent sensitivity (response time of 99 s), indicating a more rapid initial sensing behavior. This rapid response may be attributed to the imine bond (-C=N-) formed through the Schiff base reaction being a strong functional group, and the pores can retain a small amount of unreacted amino groups (-NH_2_) and aldehyde groups (-CHO), thereby exposing more active sites for ammonia adsorption. To further evaluate the gas sensing performance of the COF sensor, we systematically tested its stability, response characteristics, and gas selectivity at room temperature.

As shown in Figure 7c, the short-term cycling stability of the sensor was tested by continuously exposing it to 100 ppm ammonia (NH_3_) five times. To further assess the stability of the response, the relative standard deviation (RSD) of the response/recovery time over five cycles was calculated (Table S4). The RSD for all five cycles was maintained within 1.6%, demonstrating excellent stability. The response characteristics at different ppm levels are another parameter of sensing performance. In this study, the ammonia sensor was placed in a room temperature environment, and the effect of concentration on sensor performance was investigated by adjusting the standard gas concentration (10 ppm, 20 ppm, 30 ppm, 40 ppm, 50 ppm, 60 ppm). The response changes in the sensor at different concentrations are shown in Figure 7d. The experimental results indicate that the gas response increases proportionally with the increase in NH_3_ concentration. Figure 7e shows the relationship between the sensor response and ammonia. The fitted equation can be used for quantitative detection of gas concentration, converting the relative signal into an absolute concentration value. It has a strong correlation (R^2^ = 0.993). The theoretical detection limit (LOD) can be calculated using Formula (1), where Standard Error represents the standard error of the sensor response, and Kslope is the slope of the calibration curve. The detection limit determination results show that the detection limit of COF for NH_3_ gas is 5 ppm.

(1) $$LOD=3\times \frac{S_{Standard\;Error}}{K_{Slope}}$$

In this context, S_Standard Error_ represents the standard deviation of the sensor’s baseline signal over a continuous 30 min period in a pure nitrogen atmosphere, while K_Slope_ denotes the slope of the linear fit curve between the sensor’s response and ammonia concentration (i.e., sensitivity). Using this method, the detection limit of this sensor for ammonia was calculated to be 5 ppm.

To further explore the potential influence and interference of different gases on the sensor, researchers also conducted tests on the COF sensor with various gases at a concentration of 100 ppm, including acetaldehyde (C_2_H_4_O), acetone (C_3_H_6_O), hydrogen (H_2_), and carbon dioxide (CO_2_). The sensor showed a particularly significant response to ammonia, followed by acetaldehyde, acetone, and hydrogen, but almost no response to carbon dioxide. This phenomenon may be attributed to the small size and strong polarity of the NH_3_ molecule, as well as the synergistic effect of multiple hydrogen bonds and acid–base interactions formed between the imine bonds (-C=N-) and residual amino groups (-NH_2_) in the COF and NH_3_. In contrast, molecules such as acetaldehyde and acetone, due to their weaker polarity and lower binding energy with the active sites of the COF, resulted in a significant decrease in response values, while non-polar or weakly polar gases such as hydrogen and carbon dioxide, due to their difficulty in forming stable bonds with the active sites, ultimately exhibited almost no response.

**Figure 8:**
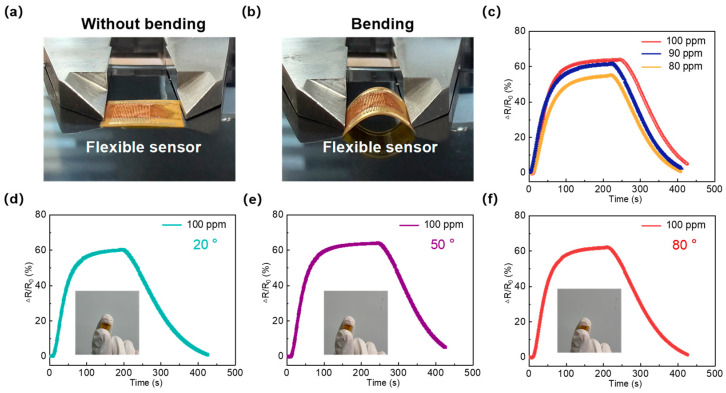
(**a,b**) The comparative testing results of the sensor in unbent and bent configurations; (**c**) gas sensing response under bending conditions at various concentrations (80 ppm, 90 ppm, 100 ppm) of Ammonia; (**d**) The response characteristics of the sensor were evaluated under bending conditions at a concentration of 100 ppm and a temperature of 20°, (**e**) 50° and (**f**) 80°.

To verify the flexibility of the fabricated sensor, we conducted a series of experiments. The flexible schematic diagram is shown in Figure S12. Firstly, Figure 8a,b show a comparison of the sensor’s morphology in its unbent and bent states, respectively. The results demonstrate that the sensor possesses excellent toughness, as no fracture occurred when bent. Figure 8c illustrates the sensor’s response characteristics to different concentrations of ammonia (80 ppm, 90 ppm, 100 ppm) under bending conditions. It can be observed that as the ammonia concentration increases, the sensor’s response value also increases. Figure 8d–f present the test results of the sensor’s response to ammonia at a concentration of 100 ppm under different bending angles (20°, 50°, 80°). The results indicate that the sensor is unaffected by external deformation and maintains stable response characteristics to ammonia at all times, demonstrating its practical value as a flexible gas sensor. Furthermore, compared with the measurement results obtained in the flat state, there were no significant differences in the response time or recovery time of the sensor under bending conditions. A detailed comparison of the data is provided in Table S5. In summary, the fabricated sensor is capable of maintaining stable sensing performance under varying degrees of bending, indicating that it holds great potential for application in the field of flexible ammonia sensing in indoor environments.

**Figure 9:**
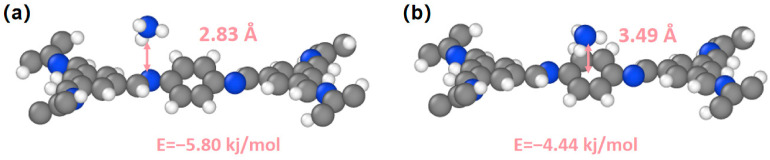
DFT simulation results for TFB-PDA-COF at different positions and with NH_3_ adsorbed; (**a**) benzene ring portion of TFB-PDA-COF; (**b**) N portion in the C=N bond of TFB-PDA-COF.

The sensing mechanism was explored by calculating the adsorption energy of NH_3_ molecules on different units of TFB-PDA-COF (Figure 9). DFT simulations were applied to study two sites on TFB-PDA-COF. One is the benzene ring structure in TFB-PDA-COF, where NH_3_ molecules adsorb on the benzene ring with a formation energy of −4.44 kJ/mol and a distance of 3.49 Å. When the site is changed to the N atom on the C=N, NH_3_ molecules adsorb with a formation energy of −5.80 kJ/mol and a distance of 2.83 Å. By comparing the two binding sites, the results are as predicted: TFB-PDA-COF is more likely to adsorb NH_3_ molecules on the N atom and provides a lower adsorption energy.

## 4. Conclusions

In summary, this study developed an innovative zinc-ion-guided solvent-free synthesis method, successfully achieving the rapid and green preparation of highly crystalline TFB-PDA-COF. The material was coated on a flexible polyimide (PI) substrate to construct an ammonia gas sensor, which demonstrated high sensitivity, fast response/recovery characteristics, and excellent selectivity to low-concentration ammonia gas at room temperature. It showed excellent cycling performance at 100 ppm NH_3_ and a strong linear response within the range of 10 ppm to 60 ppm NH_3_ concentration. This work not only provides a new paradigm for the green and controllable synthesis of covalent organic frameworks but also lays a solid material and technical foundation for the development of next-generation high-performance flexible gas sensor devices.

## Data Availability

The original contributions presented in this study are included in the article/Supplementary Material. Further inquiries can be directed to the corresponding authors.

## Supplementary Materials

The following supporting information can be downloaded at: https://www.mdpi.com/article/10.3390/mi17040499/s1, Figure S1: Schematic diagram of XRD analysis of reactions using solid Zn(OTf)_2_ and liquid Zn(OTf)_2_ as catalysts; Figure S2: SEM image of the product obtained following the reaction using a liquid Zn(OTf)_2_ catalyst; Figure S3: Schematic diagram of the EDS analysis of the products obtained following the reaction using the liquid Zn(OTf)_2_ catalyst; Figure S4: XRD at different stages of the reaction; Figure S5: BET analysis (a) and pore size distribution (b) of TPB-PDA-COF; Figure S6: SEM of TFB-PDA COFZn; Figure S7: Energy-Dispersive X-ray Spectroscopy (EDS) spectrum of TFB-PDA COF; Figure S8: Energy-Dispersive X-ray Spectroscopy (EDS) spectrum of TFB-PDA COFZn; Figure S9: X-ray Photoelectron Spectroscopy (XPS) spectrum of TFB-PDA-COF (a) C 1s XPS spectrum of TFB-PDA-COF; (b) N 1s XPS spectrum of TFB-PDA-COF; Figure S10: Schematic diagram of the ammonia gas sensing test platform; Figure S11: Schematic diagram of an ammonia sensing device using TFB-PDA-COF deposited on PI substrate; Figure S12: Schematic Illustration of Flexible Ammonia Sensing via TFB-PDA-COF deposited on PI substrate. Table S1: (100) half-peak width of TFB-PDA-COF prepared using catalysts with different concentrations; Table S2: (100) half-peak width of TFB-PDA-COF prepared at different temperatures; Table S3: Summary of NH3 gas sensors based on MOFs and COFs at room temperature; Table S4: Response values for 5 cycles; RSD of response/recovery time; Table S5: Statistical results on performance in bent and flat conditions; Video S1: LZU-1 water washing process. References [40,41,42,43] are cited in the supplementary materials.
