# Supplementary material for: Solvent-Free Synthesis of Covalent Organic Frameworks for High-Performance Room Temperature Ammonia Sensing
Source: Micromachines (Basel). 2026 Apr 20;17(4):499. doi: 10.3390/mi17040499 (PMC13118953; doi:10.3390/mi17040499)
Supplement: Supplementary file 1 [file micromachines-17-00499-s001.zip › Supporting Information.pdf]

Supplementary Materials

1. Materials

1,3,5-Benzenetricarboxaldehyde(TFB),p-Phenylenediamine(PDA),1,3,5-Tris(p-formylphenyl)benzene (TFPB), and Zinc(II) p-toluenesulfonate ($Zn(OTf)_2$) were purchased from Aladdin Industrial Corporation. All reagents were of analytical grade and used directly without further purification.

2. Computational Simulations

The adsorption of ammonia molecules onto organic structures was simulated utilizing the Materials Studio software package, with corresponding formation energies and distances calculated for various unit configurations during the adsorption process.

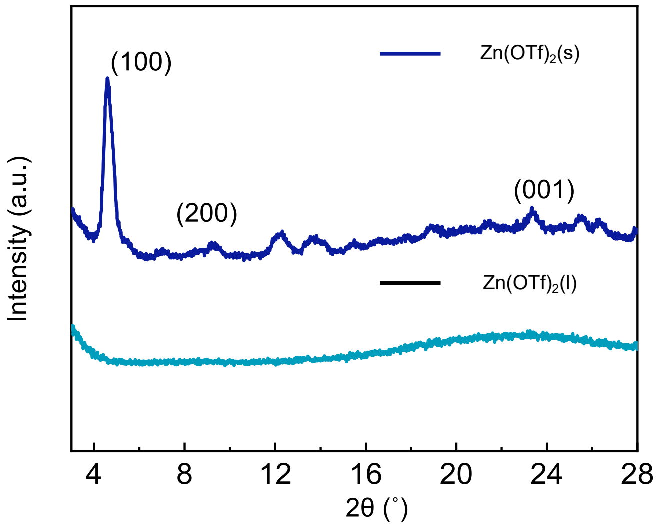

Figure S1. Schematic diagram of XRD analysis of reactions using solid $Zn(OTf)_2$ and liquid $Zn(OTf)_2$ as catalysts

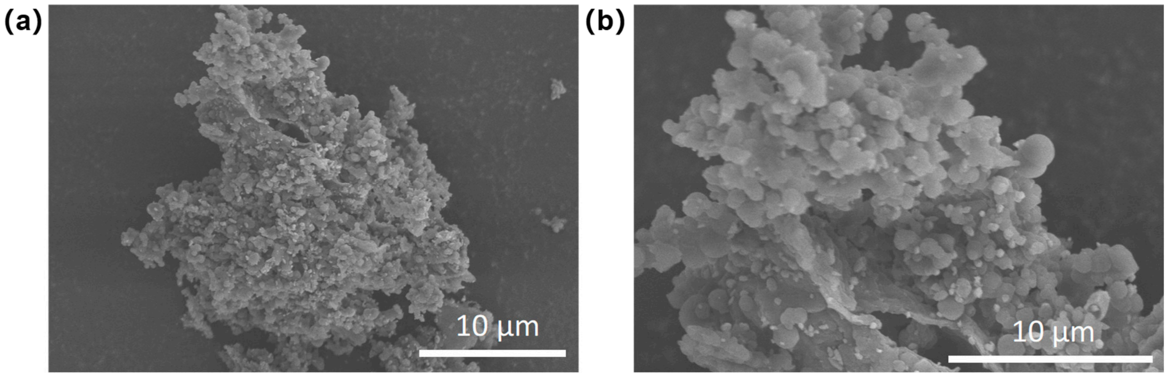

Figure S2. SEM image of the product obtained following the reaction using a liquid $Zn(OTf)_2$ catalyst

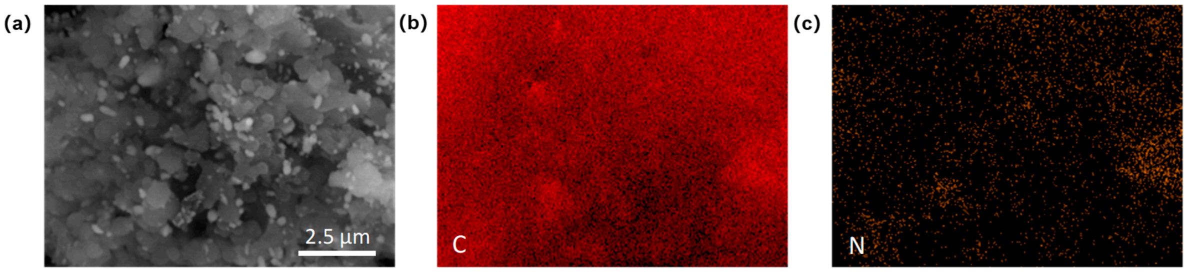

Figure S3. Schematic diagram of the EDS analysis of the products obtained following the reaction using the liquid $Zn(OTf)_2$ catalyst

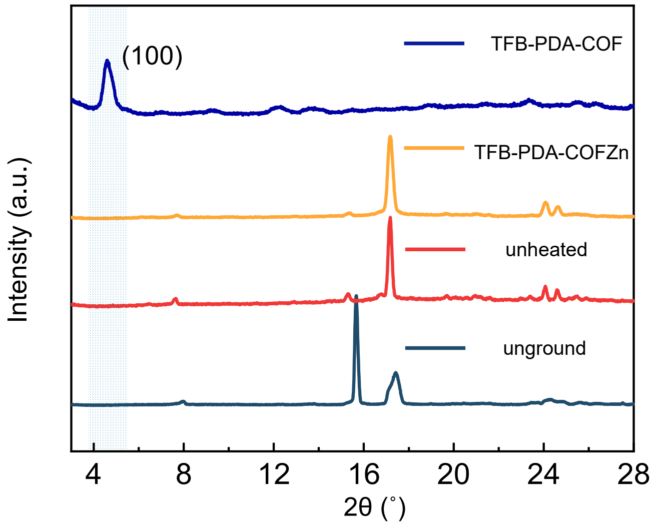

Figure S4. XRD at different stages of the reaction

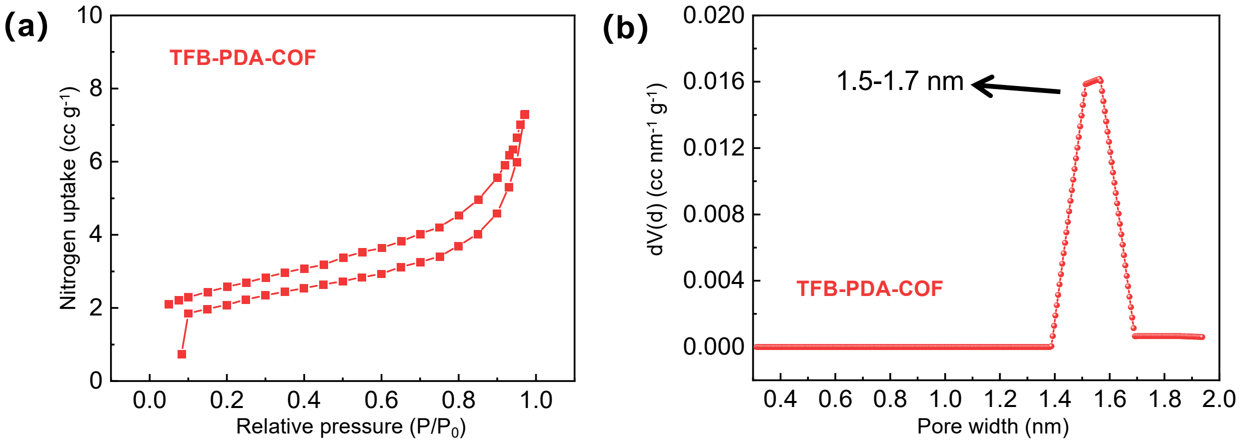

Figure S5. BET analysis (a) and pore size distribution (b) of TPB-PDA-COF

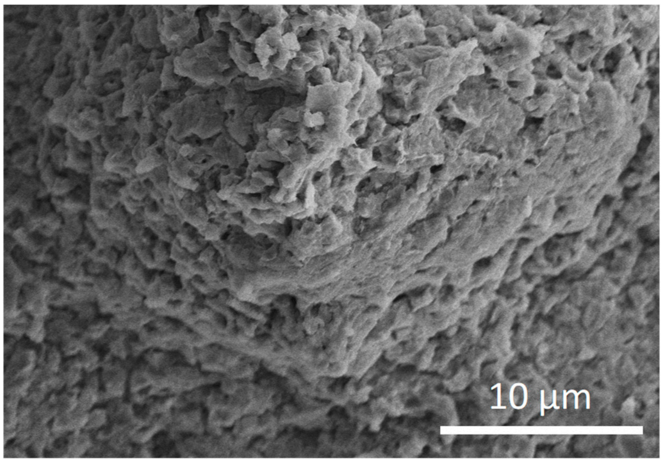

Figure S6. SEM of TFB-PDA COFZn

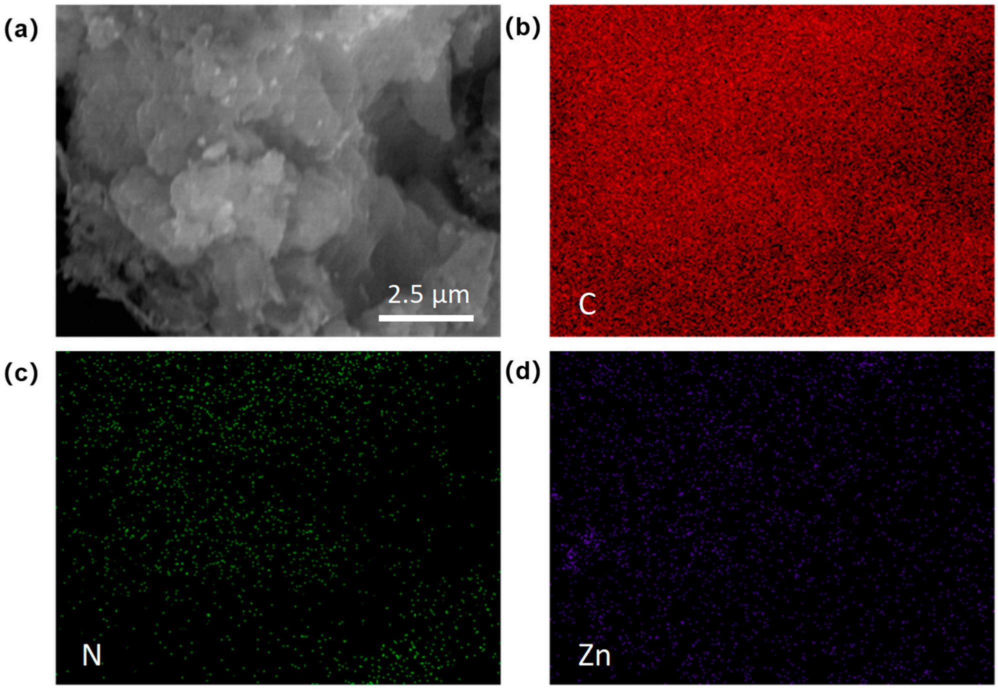

Figure S7. Energy-Dispersive X-ray Spectroscopy (EDS) spectrum of TFB-PDA COF

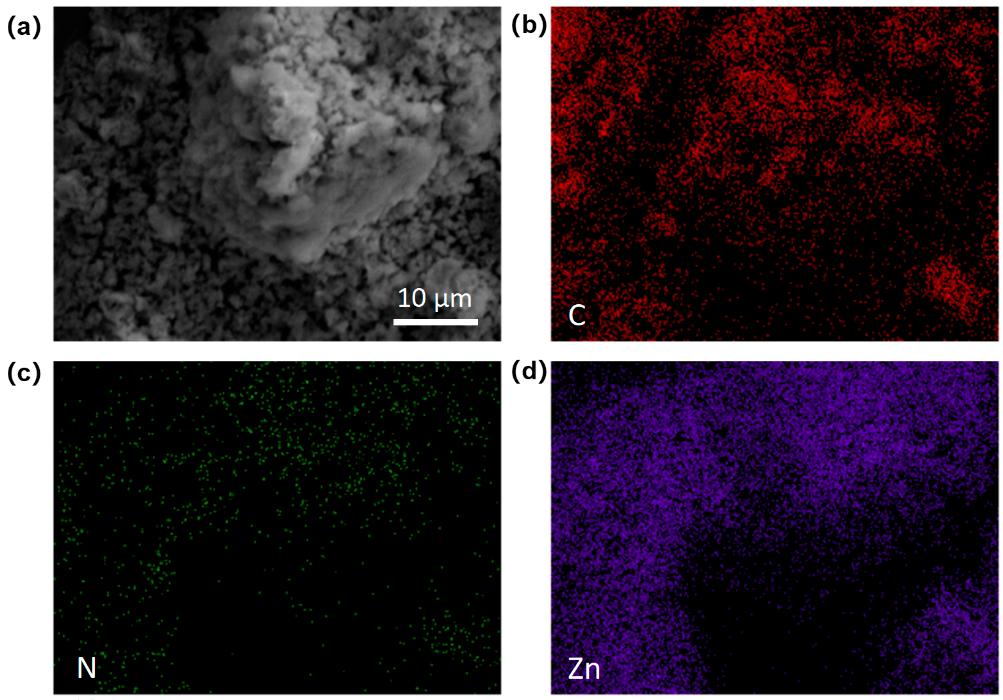

Figure S8. Energy-Dispersive X-ray Spectroscopy (EDS) spectrum of TFB-PDA COFZn

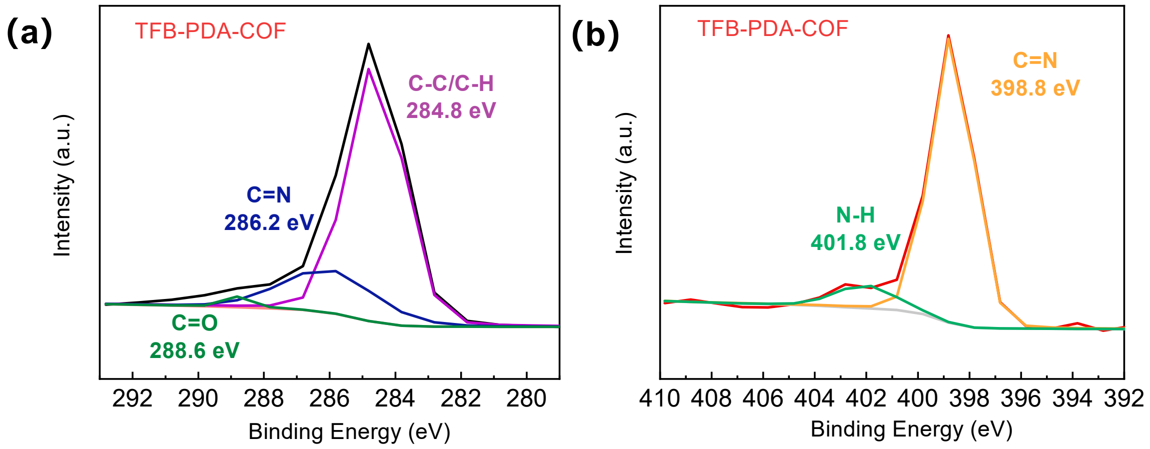

Figure S9. X-ray Photoelectron Spectroscopy（XPS）spectrum of TFB-PDA-COF (a) C 1s XPS spectrum of TFB-PDA-COF; (b) N 1s XPS spectrum of TFB-PDA-COF

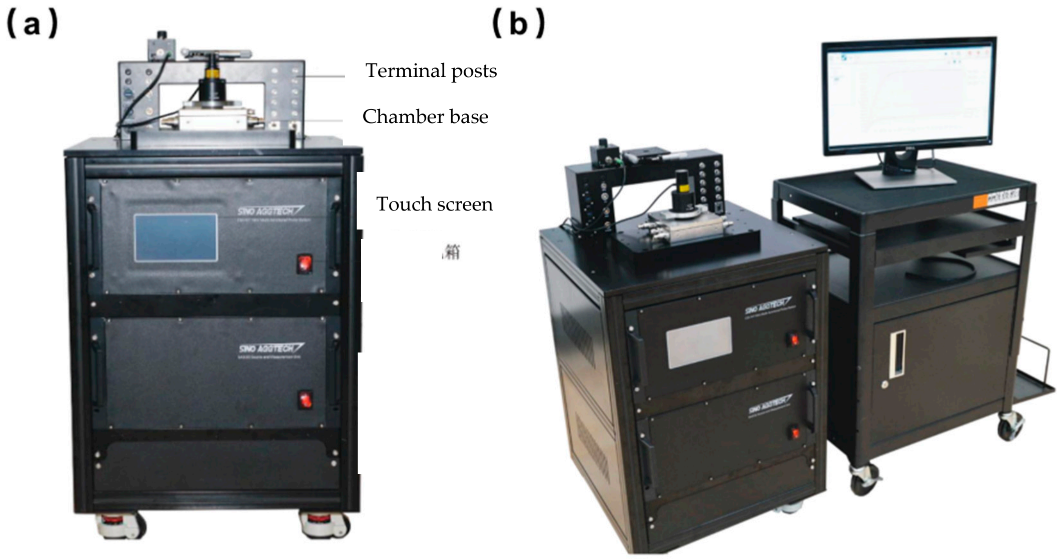

Figure S10. Schematic diagram of the ammonia gas sensing test platform.

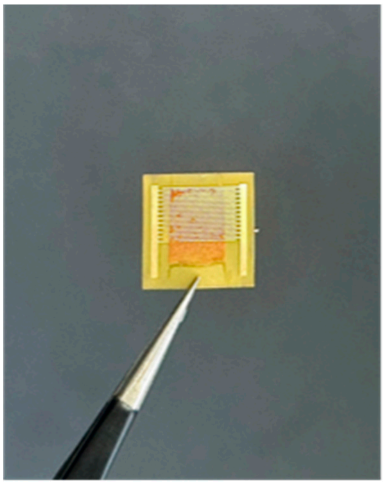

Figure S11. Schematic diagram of an ammonia sensing device using TFB-PDA-COF deposited on PI substrate.

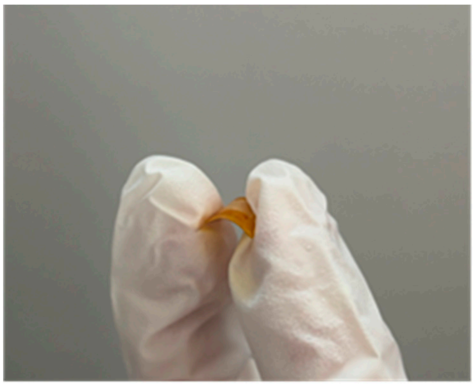

Figure S12. Schematic Illustration of Flexible Ammonia Sensing via TFB-PDA-COF deposited on PI substrate.

Table S1. (100) half-peak width of TFB-PDA-COF prepared using catalysts with different concentrations

**TFB:Zn(OTf)$_2$ mass ratio**	**1:3**	**1:6**	**1:9**	**1:12**	**1:15**
(100)half band width	0.57	0.46	0.54	0.65	0.76

Table S2. (100) half-peak width of TFB-PDA-COF prepared at different temperatures

**Temperature(℃)**	**60**	**80**	**100**	**120**	**140**
(100)half band width	-	0.95	0.65	0.48	0.56

Table S3. Summary of NH_3 gas sensors based on MOFs and COFs at room temperature

**Items**	**COFs (metallized)**	**Response value**	**Response/Recovery Speed**
[40]	TAPB-BPDA COF	-23 %（100 ppm）	10/106 s
[41]	$Cu_3(HITP)_2$	2.5 % (10 ppm)	\
[42]	Cu-HHTP-THQ	~ 20 % (100 ppm)	99/154.2 s
[43]	NiNi-Pyz	~8 % (1000 ppm)	5/55 s
\	This work	60 %（100 ppm）	99/176 s

Table S4. Response values for 5 cycles; RSD of response/recovery time

**Core parameters**	**Mean ± Standard Deviation**	**Relative standard deviation (RSD)**
Response value $\Delta R/R_0$(%)	60.7±0.4%	0.61 %
Response time	99±1.6 s	1.60 %
Recovery time	176±1.6 s	0.90 %

Table S5. Statistical results on performance in bent and flat conditions

**Test status**	**100 ppm NH_3 response value (%)**	**Response time (s)**	**Recovery time (s)**	**Performance deviation from flat state**
Flat state (no bending)	60	99	176	/
Bent 20°	60.3	102	179	Response value deviation: +0.5 %, response time deviation: +3.0 %

Bent 50°	61	105	182	Response value deviation: 1.6 %, response time deviation: +6.1 %
Bent 80°	60.7	108	185	Response value deviation: 1.1 %, response time deviation: +9.1 %
